# Migration and allergic diseases: Findings from a population‐based study in adults in Amsterdam, the Netherlands

**DOI:** 10.1111/all.15427

**Published:** 2022-07-21

**Authors:** Abena S. Amoah, Maria Prins, Elisabeth H. D. Bel, Wytske J. Fokkens, Aeilko H. Zwinderman, Maria Yazdanbakhsh, Anke H. Maitland‐van der Zee, Ronald van Ree

**Affiliations:** ^1^ Department of Parasitology Leiden University Medical Center Leiden the Netherlands; ^2^ Department of Population Health, Faculty of Epidemiology and Population Health London School of Hygiene and Tropical Medicine London UK; ^3^ Malawi Epidemiology and Intervention Research Unit Chilumba Malawi; ^4^ Department of Infectious Diseases Public Health Service of Amsterdam Amsterdam the Netherlands; ^5^ Department of Internal Medicine, Division of Infectious Diseases Amsterdam University Medical Centers, location AMC Amsterdam the Netherlands; ^6^ Department of Respiratory Medicine Amsterdam University Medical Centers, location AMC Amsterdam the Netherlands; ^7^ Department of Otorhinolaryngology Amsterdam University Medical Centers, location AMC Amsterdam the Netherlands; ^8^ Department of Clinical Epidemiology, Biostatistics and Bioinformatics Amsterdam University Medical Centers, location AMC Amsterdam the Netherlands; ^9^ Department of Experimental Immunology Amsterdam University Medical Centers, location AMC Amsterdam the Netherlands

To the Editor,

Although international migration from low‐ and middle‐income countries to high‐income countries is associated with changing exposure to allergy risk factors, its role in allergic disease manifestation is understudied.^1^ We investigated migration‐related factors and doctor‐diagnosed allergic diseases among adults from five ethnic groups living in Amsterdam, the Netherlands enrolled in the population‐based “Healthy Life in an Urban Setting” (HELIUS) study^2^, ^3^ (see Methodology in Supplementary Material). Migration‐related factors were “migrant generation” (second‐generation ethnic minority versus first‐generation ethnic minority), “age at the time of migration” and “residence duration in the Netherlands.” Information on allergies, migration‐related factors and other parameters was collected by questionnaire and physical examination. The descriptive analysis explored whether allergic disease prevalence differed between groups using Pearson's χ^2^ tests. Logistic regression examined associations between migration‐related factors and outcomes (outcomes detailed in Table S1) with results presented as adjusted odds ratios (aORs) with 95% confidence intervals (CIs).

Data were analysed from 21,850 individuals of Dutch (*n* = 4564, 20.9%), Surinamese (*n* = 7427, 34.0%), Turkish (*n* = 3614, 16.6%), Moroccan (*n* = 3906, 17.9%) and Ghanaian (*n* = 2339, 10.7%) origin. A total of 13,451 (61.6%) participants were first‐generation ethnic minorities and 3835 (17.6%) were second‐generation.

Table S2 shows participant characteristics stratified by Dutch‐origin, first‐generation and second‐generation groups.

Significant differences in the prevalence of allergy outcomes were observed (Figures 1 and S1). Nasal allergy was reported among 22.7% of participants and was significantly higher in the second‐generation (27.1%) and first‐generation (22.1%) groups compared to the Dutch‐origin group (20.5%). Asthma was prevalent in 10.5% and significantly higher in both second‐generation (14.2%) and first‐generation (10.1%) participants compared to Dutch‐origin participants (8.6%). Model covariates for multivariate analysis are shown in Table S3. As shown in Table 1, being from the second‐generation (compared to first‐generation) was significantly associated with nasal allergy (aOR = 1.15, 95% CI 1.02–1.31, *p* = .025), asthma (aOR = 1.82, 95% CI 1.54–2.15, *p* < .001) and eczema in the past 12 months (aOR = 1.28, 95% CI 1.06–1.54, *p* = .010) but not with food allergy (aOR = 1.22, 95% CI 0.99–1.51, *p* = .067) or chronic rhinosinusitis (aOR = 0.88, 95% CI 0.69–1.12, *p* = .304).

**FIGURE 1:**
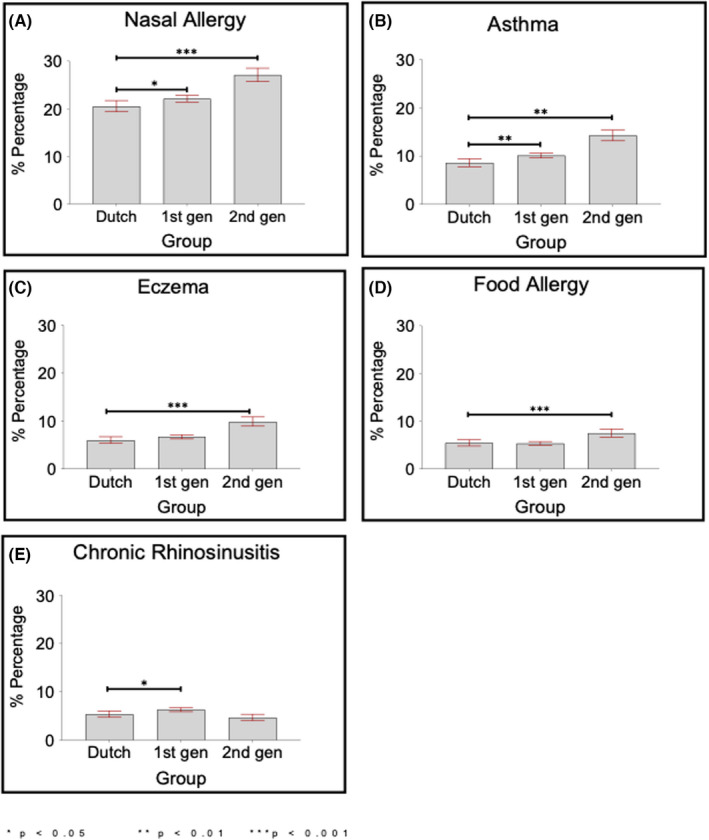
Prevalence of reported doctor‐diagnosed outcomes stratified by the Dutch‐origin group and migrant generation for the ethnic minority group. (A–E) Shows the prevalence of reported doctor‐diagnosed outcomes stratified by the Dutch‐origin group, first‐generation ethnic minorities and second‐generation ethnic minorities. Bars represent the percentage positive for each outcome and 95% CIs are also shown. *p‐*values indicate the results of Pearson's χ^2^ tests comparing the percentage positive for each outcome in the Dutch‐origin group to the two migrant generation groups, *p < 0.05, **p < 0.01, ***p < 0.001. Gen, Generation

**TABLE 1:** Results of adjusted logistic regression models exploring the associations between migration‐related factors (main exposures) and reported doctor‐diagnosed study outcomes

Main exposures (migration‐related factors)	Reported doctor‐diagnosed outcomes									
	Nasal allergy		Asthma		Eczema		Food allergy		Chronic rhinosinusitis	
	aOR (95% CI)	*p*‐value	aOR (95% CI)	*p*‐value	aOR (95% CI)	*p*‐value	aOR (95% CI)	*p*‐value	aOR (95% CI)	*p*‐value
Migrant generation (second‐generation vs first‐generation)	**1.15 (1.02–1.31)**	**0.025**	**1.82 (1.54–2.15)**	**<0.001**	**1.28 (1.06–1.54)**	**0.010**	1.22 (0.99–1.51)	0.067	0.88 (0.69–1.12)	0.304
Age at time of migration (in years)	**0.99 (0.98–0.99)**	**0.002**	**0.99 (0.98–0.99)**	**0.003**	0.99 (0.98–1.00)	0.069	1.00 (0.99–1.02)	0.371	1.00 (0.99–1.01)	0.863
Residence duration (in years)	**1.01 (1.00–1.02)**	**0.002**	**1.01 (1.00–1.02)**	**0.004**	1.01 (0.99–1.02)	0.051	0.99 (0.98–1.01)	0.361	1.00 (0.99–1.01)	0.794

Abbreviations: 95% CI, confidence interval; aOR, adjusted odds ratio.

Bold values indicate associations that were statistically significant (*p* < 0.05).

Among first‐generation participants, increasing age at the time of migration was inversely associated with nasal allergy (aOR = 0.99, 95% CI 0.98–0.99, *p* = .002) and asthma (aOR = 0.99, 95% CI 0.98–0.99, *p* = .003) but not with eczema (aOR = 0.99, 95% CI 0.98–1.00, *p* = .069), food allergy (aOR = 1.00, 95% CI (0.99–1.02, *p* = .371) or chronic rhinosinusitis (aOR = 1.00, 95% CI 0.99–1.01, *p* = .863). Conversely, longer residence duration in the Netherlands was significantly linked to nasal allergy (aOR = 1.01, 95% CI 1.00–1.02, *p* = .002) and asthma (aOR = 1.01, 95% CI 1.00–1.02, *p* = .004), eczema (OR = 1.01, 95% CI 0.99–1.02, *p* = .051—borderline) but not food allergy (aOR = 0.99, 95% CI 0.98–1.01, *p* = .361) or chronic rhinosinusitis (aOR = 1.00, 95% CI 0.99–1.01, *p* = .794).

We found that nasal allergy and asthma were significantly more prevalent among first‐ and second‐generation ethnic minority groups compared to Dutch‐origin participants. Being from the second‐generation compared to the first‐generation was significantly associated with more nasal allergy, eczema and asthma. This is consistent with other studies showing a higher allergy burden among individuals born to foreign‐born parents compared to both host populations as well as first‐generation individuals.^4^, ^5^

“Residence duration” was a proxy for exposure to environmental, lifestyle and sociocultural factors in the Netherlands that may predispose toward allergies and longer residence duration was observed to be associated with nasal allergy and asthma. Additionally, increasing age at the time of migration was inversely associated with nasal allergy and asthma suggesting that older migrants may have had greater exposure to protective factors in their countries of origin prior to migration. Comparing the epigenomic profiles of migrant and host populations could demonstrate how migration‐related changes in environmental exposures may impact disease risk.^6^

Our investigation had some limitations such as the cross‐sectional design which meant that casual associations could not be delineated. Additionally, the use of self‐reported questionnaire data may have introduced information bias and the retrospective collection of risk factors, recall bias. Hence, misclassification of both the exposure parameters (migration‐related factors) and study outcomes may have been present. Moreover, the diagnoses of our study outcomes were based on a limited number of questions.

Despite limitations, our findings indicate that intercontinental migration is positively associated with allergies among ethnic minority groups in the Netherlands. Future studies should explore these associations in other large ethnic minority groups resident in the Netherlands such as individuals of Indonesian, German or Polish ethnic origin. Studies must also investigate underlying mechanisms such as changes in the epigenome, microbiome and systemic inflammation associated with migration.

## AUTHOR CONTRIBUTION

MP, EHDB, WJF and AHZ were part of the conception, design and data acquisition for the HELIUS study. MP, AHMZ and RR were involved in the conception of the current analysis. ASA performed the analysis and all authors contributed to the interpretation of the data. ASA drafted the manuscript with input from MY and RR. All authors revised the manuscript critically and approved the final version.

## FUNDING INFORMATION

The HELIUS study is conducted by the Amsterdam University Medical Centers, location AMC and the Public Health Service of Amsterdam. Both organizations provided core support for HELIUS. The HELIUS study is also funded by the Dutch Heart Foundation (Grant number: 2010T084 [K Stronks]), the Netherlands Organization for Health Research and Development—ZonMw (Grant number: 200500003 [K Stronks]), the European Union (FP‐7 ‐ 278901 [K Stronks]) and the European Fund for the Integration of Third‐Country Nationals‐EIF (2013EIF013 [K Stronks]).

## CONFLICT OF INTEREST

Professor Elisabeth H.D. Bel reports research grants paid to her institution from GlaxoSmithKline and Teva, and personal fees for consulting from GlaxoSmithKline, AstraZeneca, Sanofi, Roche and Teva.

Professor Wytske J. Fokkens received consultation and/or speaker fees from Bioinspire, GSK, Novartis and Sanofi‐Aventis/Regeneron. The department of Otorhinolaryngology of Amsterdam University Medical Centre (location AMC) received grants for research in Rhinology from ALK, Allergy Therapeutics, Chordate, Novartis, EU, GSK, MYLAN, Sanofi‐Aventis and Zon‐MW.

Professor Anke H. Maitland‐van der Zee is the principal investigator of a P4O2 (Precision Medicine for more Oxygen) public‐private partnership sponsored by Health Holland involving many private partners that contribute in cash and/or in kind (Boehringer Ingelheim, Breathomix, Fluidda, Ortec Logiqcare, Philips, Quantib‐U, Smartfish, SODAQ, Thirona, TopMD and Novartis). Furthermore, Professor Maitland‐van der Zee has received unrestricted research grants from GSK, Boehringer Ingelheim and Vertex, and has had received consulting fees paid to her institution from Boehringer Ingelheim and AstraZeneca. She has also received honoraria for lectures paid to her institution from GlaxoSmithKline; outside the submitted work.

Professor Ronald van Ree has consultancies with HAL Allergy BV, Citeq BV, Angany Inc., Reacta Healthcare Ltd., Mission MightyMe and AB Enzymes and has received speaker's fees from HAL Allergy BV, ThermoFisher Scientific, ALK, as well as stock options with Angany Inc.

## Supporting information


Figure S1

Appendix S1

Table S1
Table S2
Table S3
