# Supplementary figures and images for: Migration and allergic diseases: Findings from a population‐based study in adults in Amsterdam, the Netherlands
Source: Allergy. 2022 Jul 21;77(12):3667–70. doi: 10.1111/all.15427 (PMC10084123; doi:10.1111/all.15427)

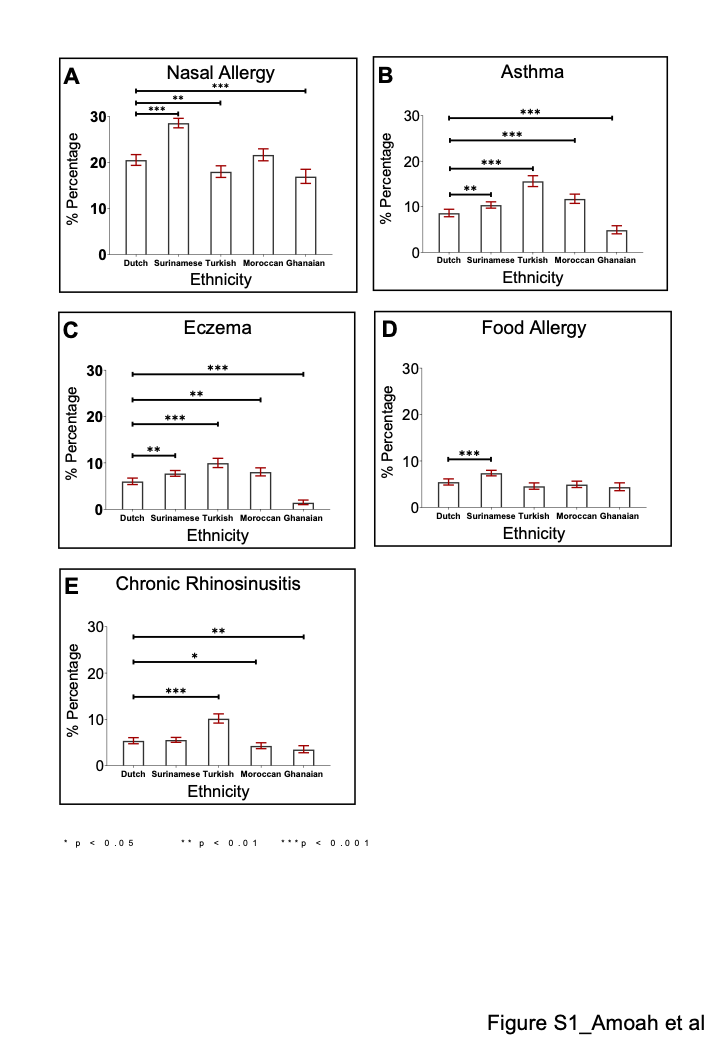

Supplement: Supplementary file 1 — Figure S1 [file ALL-77-3667-s003.tif]
