# Supplementary material for: Migration and allergic diseases: Findings from a population‐based study in adults in Amsterdam, the Netherlands
Source: Allergy. 2022 Jul 21;77(12):3667–70. doi: 10.1111/all.15427 (PMC10084123; doi:10.1111/all.15427)
Supplement: Supplementary file 2 — Appendix S1 [file ALL-77-3667-s002.docx]

Migration and allergic diseases: findings from a population-based study in adults in Amsterdam, the Netherlands

# Supplementary Material

# Methods

### Study Population

The work described here was part of baseline data collection for the population-based HELIUS study on health outcomes among five ethnic groups living in Amsterdam, the Netherlands and was conducted between January 2011 and December 2015. The HELIUS study protocol and population have been described in detail elsewhere.^1^

Briefly, the city of Amsterdam municipal register was used to randomly sample potential study participants aged between 18 and 70 years stratified by ethnicity. ‘Ethnicity’ was defined by the country of birth of potential participants and their parents.^2^ Countries of origin for the investigation were the Netherlands, Suriname, Turkey, Morocco and Ghana. The Surinamese group was further classified according to self-reported racial background and categorised as ‘South-Asian Surinamese’, ‘African Surinamese’, ‘Javanese Surinamese’ and ‘other/unknown Surinamese’ (participants who listed Suriname as a country of origin but did not specify a racial background).

For the HELIUS investigation, 90,019 individuals were sent a written invitation to participate. Of the initial 90,019 contacted, 55% (N=49,952) gave a response either to the initial invitation or a subsequent home visit. Of these 50% (24,789 / 49,952) enrolled in the study. A total of 23,942 participants completed a questionnaire on general health, specific illnesses, socioeconomic and demographic factors, lifestyle, migration history and acculturation.^1^

### Informed Consent and Ethical Approval

The HELIUS Study was approved by the Institutional Review Board of the Academic Medical Center of the University of Amsterdam (METC approval number 10/100, NL32251.018.10) and written informed consents were obtained from participants.

### Outcomes: Allergic Diseases

Our study outcomes are shown in Table S1. For most of the allergy outcomes, the questionnaire specifically asked whether a doctor had ever diagnosed the outcome. The question on eczema asked about eczema in the preceding 12 months and whether this was diagnosed by a doctor. For our analysis, we only considered ‘doctor-diagnosed eczema’ as a positive response while eczema not diagnosed by a doctor was coded as missing to exclude these individuals from the analysis. Although not an allergic disease, chronic rhinosinusitis was included in our analysis due to its close link to nasal inflammation. No distinction was made in the questionnaire between allergic and non-allergic asthma. Therefore, ‘asthma’ in our analysis included all sub-types. Although we tried to minimise potential misclassification by being more specific and focusing only on self-reported doctor-diagnosed allergic disease, this may have underestimated allergic disease in study participants who may have had limited access to healthcare or lower levels of health-care seeking behaviour.

### Explanatory variables: Migration-related factors

A participant was defined as being of non-Dutch ethnic origin if they were either [1] born outside of the Netherlands and had at least one parent born outside the Netherlands (first-generation) or [2] born in the Netherlands but both parents were born outside (second-generation). The Dutch-origin sample consisted of individuals born in the Netherlands whose parents were also born in the Netherlands. Information on residence duration and age at the time of migration to the Netherlands was also collected by questionnaire. Our main explanatory variables were:

1. Migrant Generation (second-generation versus first-generation).
2. Age at the time of migration to the Netherlands (continuous variable) – for first-generation ethnic minorities only.
3. Residence duration in the Netherlands (continuous variable) – for first-generation ethnic minorities only.

### Covariates: Socioeconomic status, Lifestyle factors and Body composition

Information on socioeconomic status (educational level), lifestyle factors (alcohol intake, smoking, physical activity) were obtained as previously described. ^1,3,4^ Physical activity was included because of its association with sedentary lifestyle and asthma development.^5^ Physical activity was measured by the SQUASH questionnaire ^6^ and defined as achieving the Dutch physical activity norm of 30 minutes on 5 or more days per week. Data on body mass index (BMI) was calculated by measuring height and weight during a physical examination while fat percentage was determined by bioelectrical impedance.^7^

### Statistical Analysis

Data analysis was performed using STATA version 15.0 (StataCorp, College Station, Texas). Outcomes were reported doctor-diagnosed diseases and the main explanatory variables were the migration-related factors. Potential confounders considered were ethnicity, demographics, socioeconomics, body composition and lifestyle factors. In descriptive analysis, we explored whether the prevalence of each outcome differed between each migrant-generation compared to the Dutch-origin group using Pearson’s χ2 tests. Logistic regression examined associations between each migration-related factor and study outcomes in three sets of models. Since residence duration and age at the time of migration were continuous variables, odds ratios (ORs) generated indicated increased or decreased odds of each reported outcome for one year increase in residence duration or one year increase in age at the time of migration. Due to multicollinearity, our main explanatory variables were not included in the same models. Unadjusted and adjusted ORs and 95% confidential intervals (CIs) were generated from univariate and multivariate logistic regression models. We performed the multivariate analysis with adjusted models that included ethnicity as a potential confounder. Potential confounders found to have a crude association with any of the outcomes in at least one ethnic group (using a cut-off of p<0.1) were retained for multivariate analysis. However, age and sex were included in all models as *a priori* confounders. All regression model covariates are shown in Table S3.

# Supplementary Material References

1. Snijder MB, Galenkamp H, Prins M, et al. Cohort profile: the Healthy Life in an Urban Setting (HELIUS) study in Amsterdam, The Netherlands. *BMJ open.* 2017;7(12):e017873.

2. Stronks K, Kulu-Glasgow I, Agyemang C. The utility of 'country of birth' for the classification of ethnic groups in health research: the Dutch experience. *Ethnicity & health.* 2009;14(3):255-269.

3. Perini W, Snijder MB, Peters RJG, Stronks K, Kunst AE. Increased cardiovascular disease risk in international migrants is independent of residence duration or cultural orientation: the HELIUS study. *J Epidemiol Community Health.* 2018;72(9):825-831.

4. Aarab R, Vijverberg SJH, Prins M, et al. Prevalence of and factors associated with adult-onset asthma in different ethnic groups: The HELIUS study. *Respiratory medicine.* 2019;150:113-119.

5. Eijkemans M, Mommers M, Draaisma JM, Thijs C, Prins MH. Physical activity and asthma: a systematic review and meta-analysis. *PLoS One.* 2012;7(12):e50775.

6. Nicolaou M, Gademan MG, Snijder MB, et al. Validation of the SQUASH Physical Activity Questionnaire in a Multi-Ethnic Population: The HELIUS Study. *PLoS One.* 2016;11(8):e0161066.

7. Meeks KA, Stronks K, Beune EJ, et al. Prevalence of type 2 diabetes and its association with measures of body composition among African residents in the Netherlands--The HELIUS study. *Diabetes research and clinical practice.* 2015;110(2):137-146.

# Tables and Figures

| **Study Outcome** | **Question** | |
| --- | --- | --- |
| Nasal Allergy | *Has a doctor ever told you that you have some form*  *of nasal (nose) allergy or hay fever?* | ☐ No ☐ Yes |
| Asthma | *Has a doctor ever told you that you have asthma?* | ☐ No ☐ Yes |
| Food Allergy | *Has a doctor ever told you that you have a food allergy?* | ☐ No ☐ Yes |
| Eczema | *Please indicate [whether you have had eczema / chronic eczema] now or that you have had it in the past 12 months, and whether or not this was diagnosed by a doctor.* | ☐ No  ☐ Yes, not diagnosed by a doctor  ☐ Yes, diagnosed by a doctor |
| Chronic Rhinosinusitis | *Has a doctor ever told you that you have chronic sinusitis (chronic infection of the sinuses) or polyps in your nose?* | ☐ No ☐ Yes |

Table S1: Disease outcomes in the questionnaire

Table S2: Characteristics of the study population stratified by Dutch-origin group and ethnic minority migrant-generation

| **Factor** | **Groups** | | | |
| --- | --- | --- | --- | --- |
|  | **Dutch-origin** | **First Generation** | **Second Generation** | **Total** |
|  | **(N=4,564)** | **(N=13,451)** | **(N = 3,835)** | **(N=21,850)** |
| **Age (years) , Mean (SD)** | 46.2 (14.1) | 48.1 (10.6) | 28.5 (7.3) | 44.3 (13.2) |
|  |  |  |  |  |
| **Sex** |  |  |  |  |
| Male | 2089 (45.8) | 5547 (41.2) | 1586 (41.4) | 9222 (42.2) |
| Female | 2475 (54.2) | 7904 (58.8) | 2249 (58.6) | 12628 (57.8) |
|  |  |  |  |  |
| **BMI, Mean (SD)** | 24.8 ( 4.2) | 28.3 (5.3) | 25.5 (5.1) | 27.1 (5.3) |
| Missing (N) | 3 | 14 | 6 | 23 |
|  |  |  |  |  |
| **Body fat percent (%), Mean (SD)** | 28.9 (7.4) | 32.9 (8.6) | 30.1 (8.2) | 31.6 (8.5) |
| Missing (N) | 92 | 265 |  | 435 |
|  |  |  |  |  |
| **Education** |  |  |  |  |
| No school/Elementary | 150 (3.3) | 3530 (26.2) | 158 (4.1) | 3838 (17.6) |
| Low vocational/second. | 646 (14.2) | 4351 (32.4) | 717 (18.7) | 5714 (26.2) |
| Intermediate vocational | 994 (21.8) | 3570 (26.5) | 1752 (45.7) | 6316 (28.9) |
| High vocational/Tertiary | 2749 (60.2) | 1854 (13.8) | 1182 (30.8) | 5785 (26.5) |
| Missing | 25 (0.6) | 146 (1.1) | 26 (0.7) | 197 (0.9) |
|  |  |  |  |  |
| **Smoking** |  |  |  |  |
| No/Never | 1689 (37.0) | 8158 (60.7) | 2331 (60.8) | 12178 (55.7) |
| Ex or current smoker | 2866 (62.8) | 5208 (38.7) | 1496 (39.0) | 9570 (43.8) |
| Missing | 9 (0.2) | 85 (0.6) | 8 (0.2) | 102 (0.5) |
|  |  |  |  |  |
| **Alcohol intake** |  |  |  |  |
| Never | 297 (6.5) | 6637 (49.3) | 1995 (52.0) | 8929 (40.9) |
| Ex or current drinker | 4261 (93.4) | 6711 (49.9) | 1829 (47.7) | 12801 (58.6) |
| Missing | 6 (0.1) | 103 (0.8) | 11 (0.3) | 120 (0.6) |
|  |  |  |  |  |
| **Achieved Dutch norm for physical activity (30 minutes on >=5 days per week)** |  |  |  |  |
| No | 1114 (24.4) | 6310 (46.9) | 2072 (54.0) | 9496 (43.5) |
| Yes | 3446 (75.5) | 7127 (53.0) | 1752 (45.7) | 12325 (56.4) |
| Missing | 4 (0.1) | 14 (0.1) | 11 (0.3) | 29 (0.1) |
|  |  |  |  |  |
| **Age at time of migration (years), Mean (SD)** | - | 19.8 (10.3) | - | 19.8 (10.3) |
| Missing (N) |  | 386 |  | 386 |
|  |  |  |  |  |
| **Residence duration (years), Mean (SD)** | - | 28.7 (10.3) | - | 28.7 (10.3) |
| Missing (N) |  | 386 |  | 386 |

Table S3: Covariates in each adjusted logistic regression model

| **Covariates** | **Adjusted Logistic Regression Models** | | | | |
| --- | --- | --- | --- | --- | --- |
|  | **Nasal Allergy** | **Asthma** | **Eczema** | **Food Allergy** | **Chronic Rhinosinusitis** |
| Nasal allergy |  | ✓ | ✓ | ✓ | ✓ |
| Asthma | ✓ |  | ✓ | ✓ | ✓ |
| Eczema | ✓ | ✓ |  | ✓ | ✓ |
| Food Allergy | ✓ | ✓ | ✓ |  | ✓ |
| Chronic Sinusitis | ✓ | ✓ | ✓ | ✓ |  |
| Ethnicity | ✓ | ✓ | ✓ | ✓ | ✓ |
| Age (years) | ✓ | ✓ | ✓ | ✓ | ✓ |
| Sex | ✓ | ✓ | ✓ | ✓ | ✓ |
| BMI, Mean | ✓ | ✓ | ✓ |  | ✓ |
| Body fat percent | ✓ | ✓ |  | ✓ | ✓ |
| Education | ✓ | ✓ | ✓ | ✓ | ✓ |
| Smoking | ✓ | ✓ | ✓ | ✓ | ✓ |
| Alcohol intake | ✓ | ✓ | ✓ |  | ✓ |
| Physical activity |  | ✓ |  |  |  |

# Figure Legend

**Figure S1: Prevalence of reported doctor-diagnosed outcomes stratified by ethnic group**

Figure S1 (A-E) shows the prevalence of reported doctor-diagnosed outcomes among the five ethnic groups investigated. Bars represent the percentage positive for each outcome and 95% Confidence Intervals (CIs) are also shown.

*p-*values indicate the results of Pearson’s χ^2^ tests comparing the percentage positive in the Dutch-origin group to other ethnic groups, * *p*<0.05, ** *p*<0.01, *** *p*<0.001
