# Supplementary material for: Migration and allergic diseases: Findings from a population‐based study in adults in Amsterdam, the Netherlands
Source: Allergy. 2022 Jul 21;77(12):3667–70. doi: 10.1111/all.15427 (PMC10084123; doi:10.1111/all.15427)
Supplement: Supplementary file 3 — Table S1 Table S2 Table S3 [file ALL-77-3667-s001.docx]

# Supplementary Material Tables and Figures

| **Study Outcome** | **Question** | |
| --- | --- | --- |
| Nasal Allergy | *Has a doctor ever told you that you have some form*  *of nasal (nose) allergy or hay fever?* | ☐ No ☐ Yes |
| Asthma | *Has a doctor ever told you that you have asthma?* | ☐ No ☐ Yes |
| Food Allergy | *Has a doctor ever told you that you have a food allergy?* | ☐ No ☐ Yes |
| Eczema | *Please indicate [whether you have had eczema / chronic eczema] now or that you have had it in the past 12 months, and whether or not this was diagnosed by a doctor.* | ☐ No  ☐ Yes, not diagnosed by a doctor  ☐ Yes, diagnosed by a doctor |
| Chronic Rhinosinusitis | *Has a doctor ever told you that you have chronic sinusitis (chronic infection of the sinuses) or polyps in your nose?* | ☐ No ☐ Yes |

Table S1: Disease outcomes in the questionnaire

Table S2: Characteristics of the study population stratified by Dutch-origin group and ethnic minority migrant-generation

| **Factor** | **Groups** | | | |
| --- | --- | --- | --- | --- |
|  | **Dutch-origin** | **First Generation** | **Second Generation** | **Total** |
|  | **(N=4,564)** | **(N=13,451)** | **(N = 3,835)** | **(N=21,850)** |
| **Age (years) , Mean (SD)** | 46.2 (14.1) | 48.1 (10.6) | 28.5 (7.3) | 44.3 (13.2) |
|  |  |  |  |  |
| **Sex** |  |  |  |  |
| Male | 2089 (45.8) | 5547 (41.2) | 1586 (41.4) | 9222 (42.2) |
| Female | 2475 (54.2) | 7904 (58.8) | 2249 (58.6) | 12628 (57.8) |
|  |  |  |  |  |
| **BMI, Mean (SD)** | 24.8 ( 4.2) | 28.3 (5.3) | 25.5 (5.1) | 27.1 (5.3) |
| Missing (N) | 3 | 14 | 6 | 23 |
|  |  |  |  |  |
| **Body fat percent (%), Mean (SD)** | 28.9 (7.4) | 32.9 (8.6) | 30.1 (8.2) | 31.6 (8.5) |
| Missing (N) | 92 | 265 |  | 435 |
|  |  |  |  |  |
| **Education** |  |  |  |  |
| No school/Elementary | 150 (3.3) | 3530 (26.2) | 158 (4.1) | 3838 (17.6) |
| Low vocational/second. | 646 (14.2) | 4351 (32.4) | 717 (18.7) | 5714 (26.2) |
| Intermediate vocational | 994 (21.8) | 3570 (26.5) | 1752 (45.7) | 6316 (28.9) |
| High vocational/Tertiary | 2749 (60.2) | 1854 (13.8) | 1182 (30.8) | 5785 (26.5) |
| Missing | 25 (0.6) | 146 (1.1) | 26 (0.7) | 197 (0.9) |
|  |  |  |  |  |
| **Smoking** |  |  |  |  |
| No/Never | 1689 (37.0) | 8158 (60.7) | 2331 (60.8) | 12178 (55.7) |
| Ex or current smoker | 2866 (62.8) | 5208 (38.7) | 1496 (39.0) | 9570 (43.8) |
| Missing | 9 (0.2) | 85 (0.6) | 8 (0.2) | 102 (0.5) |
|  |  |  |  |  |
| **Alcohol intake** |  |  |  |  |
| Never | 297 (6.5) | 6637 (49.3) | 1995 (52.0) | 8929 (40.9) |
| Ex or current drinker | 4261 (93.4) | 6711 (49.9) | 1829 (47.7) | 12801 (58.6) |
| Missing | 6 (0.1) | 103 (0.8) | 11 (0.3) | 120 (0.6) |
|  |  |  |  |  |
| **Achieved Dutch norm for physical activity (30 minutes on >=5 days per week)** |  |  |  |  |
| No | 1114 (24.4) | 6310 (46.9) | 2072 (54.0) | 9496 (43.5) |
| Yes | 3446 (75.5) | 7127 (53.0) | 1752 (45.7) | 12325 (56.4) |
| Missing | 4 (0.1) | 14 (0.1) | 11 (0.3) | 29 (0.1) |
|  |  |  |  |  |
| **Age at time of migration (years), Mean (SD)** | - | 19.8 (10.3) | - | 19.8 (10.3) |
| Missing (N) |  | 386 |  | 386 |
|  |  |  |  |  |
| **Residence duration (years), Mean (SD)** | - | 28.7 (10.3) | - | 28.7 (10.3) |
| Missing (N) |  | 386 |  | 386 |

Table S3: Covariates in each adjusted logistic regression model

| **Covariates** | **Adjusted Logistic Regression Models** | | | | |
| --- | --- | --- | --- | --- | --- |
|  | **Nasal Allergy** | **Asthma** | **Eczema** | **Food Allergy** | **Chronic Rhinosinusitis** |
| Nasal allergy |  | ✓ | ✓ | ✓ | ✓ |
| Asthma | ✓ |  | ✓ | ✓ | ✓ |
| Eczema | ✓ | ✓ |  | ✓ | ✓ |
| Food Allergy | ✓ | ✓ | ✓ |  | ✓ |
| Chronic Sinusitis | ✓ | ✓ | ✓ | ✓ |  |
| Ethnicity | ✓ | ✓ | ✓ | ✓ | ✓ |
| Age (years) | ✓ | ✓ | ✓ | ✓ | ✓ |
| Sex | ✓ | ✓ | ✓ | ✓ | ✓ |
| BMI, Mean | ✓ | ✓ | ✓ |  | ✓ |
| Body fat percent | ✓ | ✓ |  | ✓ | ✓ |
| Education | ✓ | ✓ | ✓ | ✓ | ✓ |
| Smoking | ✓ | ✓ | ✓ | ✓ | ✓ |
| Alcohol intake | ✓ | ✓ | ✓ |  | ✓ |
| Physical activity |  | ✓ |  |  |  |
